# Progression significance for low‐level aberrant B‐lymphoblasts in chronic myeloid leukaemia patients

**DOI:** 10.1002/ctm2.70524

**Published:** 2025-11-05

**Authors:** Haoren Wang, Huijun Wang, Yuanyuan Li, Bin Zheng, Qiuyun Fang, Yan Li, Yuntao Liu, Runxia Gu, Ying Wang, Yingchang Mi, Bingcheng Liu, Hui Wei, Jianxiang Wang

**Affiliations:** ^1^ National Clinical Research Center for Blood Diseases State Key Laboratory of Experimental Hematology Haihe Laboratory of Cell Ecosystem Institute of Hematology & Blood Diseases Hospital Chinese Academy of Medical Sciences & Peking Union Medical College Tianjin China; ^2^ Tianjin Institutes of Health Science Tianjin China

1

Dear Editor,

Chronic myeloid leukaemia (CML) patients with progression events face limited treatment response and dismal survival.^1^ However, the 2022 World Health Organization leaves an ambiguous threshold of the rate of lymphoblasts for blast crisis.^2^ Otherwise, several studies reported conflicting outcomes for patients with low levels of aberrant B‐lymphoblasts (ABLB), with El Rassi et al. and Vijayasekharan et al. reporting that low levels of ABLB associated with rapid progression to blast crisis,^3^, ^4^ while Vrotsos et al. and Soma et al. observing sustained deep molecular responses under tyrosine kinase inhibitor (TKI) therapy.^5^, ^6^ To explore the prognostic significance of low levels of ABLB, this study systematically evaluated the clinical characteristics and treatment response of 16 patients with low levels of ABLB and determined the cutoff value of ABLB associated with disease progression, providing a new risk factor for progression in CML patients and optimising risk‐adapted therapeutic decisions.

Among 802 chronic phase‐CML (CP‐CML) patients who underwent flow cytometry (FCM) analysis at the time of diagnosis, 16 (2.0%) had a low level of ABLB, which was defined as <5%, in line with the 2022 International Consensus Classification (ICC) guideline that morphologically apparent lymphoblasts >5% should prompt consideration of lymphoblastic crisis.^7^ The level of ABLB ranges from  .01% to 4.69%, and 10 of them experienced progression events. Of these 16 patients, eight were male, and their ages ranged from 15 to 76 years. Only one patient (case 7), a 57‐year‐old male, was classified as high‐risk according to Sokal and EUTOS long‐term survival (ELTS) scores. No additional high‐risk factors revealed for the drug resistance or progression occurring (Table S1). The immunophenotype of abnormal cells is shown in Table S2. Particular attention should be paid to B progenitors in FCM reports, as aberrant B lymphoblasts may exhibit immunophenotypic features closely resembling those of B progenitors, making them easily confused.

These 16 patients were included as a training group for the analysis of the cutoff value of ABLB associated with progression, and 18 patients from five reported studies^3^, ^4^, ^5^, ^6^, ^8^ were included as a validation group (ABLB levels range from  .006% to 3.4%; Table S3). Among the total of 34 patients, those with progression events had higher levels of ABLB compared to those without progression events (*p *= .0225; Figure 1A). The area under the ROC curve was 86.4912 (Figure 1B), indicating a significant association between ABLB levels and the risk of progression. The cutoff value for ABLB was confirmed as  .2% by the surv_cutpoint function. Patients with ≤.2% ABLB exhibited significantly longer progression‐free survival than those with higher ABLB levels in the training group (*p *= .011; Figure 1C) and validation group (*p *= .044; Figure 1D), respectively.

**FIGURE 1:**
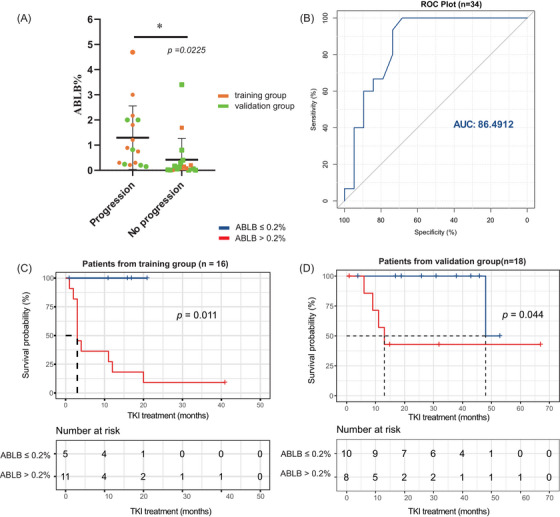
Level of aberrant B‐lymphoblasts (ABLB) associated with the risk of progression. (A) Patients with progression events had a higher level of ABLB; yellow points represent patients from this study, and green points represent patients from previously reported studies. (B) Area under the ROC curve (AUC) for ABLB was 86.4616, indicating a strong association between ABLB levels and the risk of progression. (C and D) Patients with ABLB ≤.2% (blue) had significantly longer progression‐free survival than patients with ABLB >.2% (red) in training group and validation group, respectively.

Considering TKI regimens is important to interpreting the prognostic value of ABLB, we further conducted a stratified Cochran‒Mantel‒Haenszel analysis to address potential confounding by treatment. The association remained significant after conditioning on TKI regimens (Mantel‒Haenszel *χ* ^2^ = 9.312, *p *= .002). The test for homogeneity of stratum‐specific odds ratios was not significant (Breslow–Day test, *p *= .176; Tarone's test, *p *= .188), indicating no observed effect modification by treatment in this dataset. The common Mantel–Haenszel odds ratio was 24.238 (95% confidence interval 2.803‒209.575; *p *= .004), indicating markedly higher odds of progression for patients with ABLB >.2% versus ≤.2%. The association between ABLB and progression stratified by TKI regimen is shown in Table S4.

The treatment regimens of 16 patients from our center were further detailed (Table 1). Among these patients, five had ABLB ≤.2%, and none of them experienced progression events. However, two encountered failure events. One of them with ABLB of  .12% had a *BCR::ABL1* level greater than 1% at the 16th month of Nilotinib treatment, the other one with ABLB of  .2% did not achieve complete cytogenetic response (CCyR) by the 21st month, suggesting that achieving satisfactory treatment responses for patients with ABLB was challenging.

**TABLE 1:** Treatment response and outcomes for CP‐CML patients with less than 5% aberrant B‐lymphoblast (ABLB).

	ABLB (%)	Treatment before progression	Progression (months)	Treatment after progression	Follow‐up (months)
ABLB ≤.2%, without progression	.01	Nilotinib, CCyR on 4th month; MMR on 11th month	None (11)	None	Alive (11)
	.08	Radotinib, lost to follow‐up	NA (1)	NA	NA
	.08	1L: Nilotinib, CCyR on 6th month; switched to Flumatinib due to pleural effusion 2L: Flumatinib, *BCR::ABL1* (IS) was .43% on 16th month	None (17)	None	Alive (17)
	.12	Nilotinib, CCyR on 3rd month Failure event: *BCR::ABL1* (IS) was 1.03% on 16th month	None (16)	None	Alive (16)
	.2	1L: Dasatinib, no CyR 2L: switched to Ponatinib due to T315I, Ph+ 65% on 21th month	None (21)	None	Alive (21)
ABLB >.2%, without progression	1.69	1L: Dasatinib, CCyR on 3rd month, but loss of CCyR on 9th month 2L: Olverembatinib + VP, underwent allo‐HSCT after achieving MMR	None (41)	None	Alive (41)
ABLB >.2%, with progression	.21	Imatinib, achieved PCyR on 3rd month, but got ph+ACA^a^ and ABL mutation (G250E; F359V) on 7th month	AP (7) BP (12)	Initial: Dasatinib due to ph+ACA, had no satisfy response and progressed into BP on 12th month Switched: Olverembatinib + VP, achieved CHR.	Died (27)
	.3	Dasatinib, no CHR and transferred into BP	BP (1)	NA	Died (1)
	.3	Imatinib, got ph+ACA^b^ on 3rd month	AP (3)	Initial: Flumatinib, CCyR on 6th month, but *BCR::ABL1* was 2.5025% on 15th month (ABL mutation was negative) Switched: Dasatinib, achieved MMR on 19th month	Alive (42)
	.75	Dasatinib, transferred into BP on 3rd month	BP (3)	NA	Alive (3)
	.89	Imatinib, transferred into BP on 3rd month	BP (3)	Olverembatinib + Blinatumomab, underwent allo‐HSCT after achieving MMR	Alive (19)
	1.21	Imatinib, transferred into BP on 2nd month	BP (2)	Dasatinib + VDCP, underwent allo‐HSCT after achieving DMR	Alive (58)
	1.8	Imatinib, no CCyR on 12th month, and transferred into BP on 20th month	BP (20)	Initial: Dasatinib for 4 months, no CHR Switched: Flumatinib + VP, achieved CCyR, but then transferred into BP again on 30th month Subsequent: Ponatinib + VMMP, no detailed response information	Died (44)
	2.17	Imatinib, transferred into BP on 4th month	BP (4)	NA	Alive (5)
	3	Flumatinib, transferred into BP on 3rd month	BP (3)	Initial: Olverembatinib + HyperCVAD, CCyR on 4th month, but detected 1.39% ABLB again on 5th month Switched: Olverembatinib + HD‐MTX + Blinatumomab, underwent allo‐HSCT after achieving MMR, but detected .18% ABLB again on 11th month Subsequent: Dasatinib + VP + Inotuzumad ozogamicin, no detailed response information	Alive (19)
	4.69	1L: Flumatinib, 1% FISH on 3rd month (normal Karyotype) 2L: switched to Dasatinib due to liver dysfunction, transferred into BP on 11th month	BP (11)	Initial: Olverembatinib + chemotherapy (no detailed), then received CAR‐T therapy, and maintained stable disease	Alive (32)

Abbreviations: allo‐HSCT, allogeneic haematopoietic stem cell transplantation; AP, accelerate phase; BP, blast phase; CCyR, complete cytogenetic response; PCyR, partial cytogenetic response; DMR, deep molecular response; MMR, major molecular response; CHR, complete hematologic response; FISH, fluorescence in situ hybridization; CAR‐T, chimeric antigen receptor T; NA, not acquired; HD‐MTX, high‐dose methotrexate; VP, Vincristine, Dexamethasone; VDCP, Vincristine, Daunorubicin, Cytarabine, Prednisone; VMMP, Vincristine, 6‐MP, MTX, Prednisone

^a^ Ph+ACA: 43, XX, add(1)(q42), add(3)(p25), add(3)(p25), ‐6, ‐7, t(9;22)(q34.1;q11.2), add(10)(q22), ‐12[4]/46, XX[26].

^b^ Ph+ACA: 43, XY, ‐6, ‐8, t(9;22)(q34.1;q11.2), ‐22[1]/46, XY[19].

Out of 11 patients with ABLB >.2%, 10 experienced progression events, and three of them died. The median time to blast transformation was 3 months, with a range of 1‒41 months from the detection of ABLB (Table 2). The only patient without progression events had an ABLB of 1.69%. This patient switched to Olverembatinib combined with VP regimens due to rapid loss of CCyR. After achieving a major molecular response (MMR), the patient underwent allogeneic haematopoietic stem cell transplantation (allo‐HSCT) and ultimately achieved long‐term disease‐free survival.

**TABLE 2:** Progression risk and treatment suggestion for patients with low levels of aberrant B‐lymphoblast (ABLB).

ABLB (%)	Risk level	Suggestion
≤.2	Not confirmed	Patients have a relatively low risk of disease progression but may experience treatment failure events; therefore, close monitoring is vital, and second‐generation TKIs are considered an optimal choice to expedite deep remission.
>.2%	High risk	Patients are at high risk of progression and show a limited response to single‐agent TKIs; therefore, TKI‐based combination therapy and allo‐HSCT should be considered.

Abbreviation: allo‐HSCT, allogeneic haematopoietic stem cell transplantation.

In this study, all ABLB assessment were performed at baseline, and most progression events occurred before the first on‐treatment molecular milestone (<3 months). We therefore position ABLB as a baseline risk marker but seemed mitigable with intensive therapy. Patients with ABLB ≤.2% demonstrated a lower incidence of progression compared to those with higher ABLB levels, yet still carried a meaningful risk of failure; accordingly, second‐generation TKIs may be optimal first‐line therapy to achieve rapid disease control. On the other hand, patients with ABLB >.2% showed limited responses to single‐agent TKIs. Thus, we recommend a combination therapy based on second‐generation TKIs, such as TKIs used in conjunction with VP regimens. Additionally, allo‐HSCT should be considered to reduce the risk of progression events (Table 2). This study is limited by a modest cohort size, which restrict statistical power and the robustness of adjusted analyses. Although we included an external cohort for transportability, the total sample remains insufficient for definitive inference. Thus, multi‐center prospective studies should be performed for the validation of these conclusions and proposals.

Overall, patients with ABLB >.2% at diagnosis had a high risk of progression. Therefore, detecting ABLB by FCM at the time of diagnosis can be a valuable process for identifying individuals with high‐risk progression to BP and improving patient outcomes through appropriate therapy.

## AUTHOR CONTRIBUTIONS

*Formal analysis, investigation and writing—original draft*: Haoren Wang. *Investigation and data curation*: Huijun Wang. *Validation*: Yuanyuan Li and Bin Zheng. *Investigation*: Qiuyun Fang, Yan Li and Yuntao Liu. *Investigation and funding acquisition*: Runxia Gu. *Validation and investigation*: Ying Wang and Yingchang Mi. *Validation, writing—review and editing, and supervision*: Bingcheng Liu. *Supervision*: Hui Wei. *Conceptualisation, funding acquisition and supervision*: Jianxiang Wang.

## ETHICS STATEMENT

This retrospective study was approved by the Ethics Committee of Institute of Hematology and Blood Diseases Hospital (IIT2019004‐EC‐1).

## Supporting information



Supporting information

Supporting information

Supporting information

Supporting information

## Data Availability

The data supporting the findings of this study are available from the corresponding author upon reasonable request.
